# Bioaccumulation of Some Heavy Metals: Analysis and Comparison of* Cyprinus carpio* and* Labeo rohita* from Sardaryab, Khyber Pakhtunkhwa

**DOI:** 10.1155/2017/5801432

**Published:** 2017-03-15

**Authors:** Ali Muhammad Yousafzai, Farhad Ullah, Fathul Bari, Sumayya Raziq, Mehreen Riaz, Khalid Khan, Umar Nishan, Iram Alam Sthanadar, Baseerat Shaheen, Mussarrat Shaheen, Habib Ahmad

**Affiliations:** ^1^Department of Zoology, Islamia College, Peshawar, Pakistan; ^2^Department of Animal Sciences, Quaid-i-Azam University, Islamabad, Pakistan; ^3^Department of Chemistry, Kohat University of Science and Technology, Kohat, KPK, Pakistan

## Abstract

We examined and compared heavy metals bioaccumulation in* Cyprinus carpio* and* Labeo rohita* netted from Sardaryab, a tributary of River Kabul. By using atomic absorption spectrometry we assessed different organs including livers, gills, and muscles. Metals studied were chromium, iron, zinc, lead, and copper. Livers of both species showed higher concentrations of metals while muscles showed the least amount. Chromium and iron were the highly concentrated metals in the gills and livers of both species. A quantity of 0.154 ± 0.011, 0.199 ± 0.0079, and 0.024 ± 0.008 *μ*g/g of chromium was found in the gills, livers, and muscles of* Cyprinus carpio*, respectively. Similarly, the gills, liver, and muscles of* Labeo rohita* contained 0.133 ± 0.008, 0.165 ± 0.01, and 0.019 ± 0.006 *μ*g/g of Cr, respectively. Iron was highest in carp in the range of 0.086 ± 0.01 in gills and 0.067 ± 0.011 *μ*g/g in muscles, comparatively. All the studied metals were found within the US recommended daily dietary allowances (RDA) limits; hence no immediate risk in their consumption for human was found. The data showed that* Cyprinus carpio* being omnivorous and bottom feeder stored higher concentrations of metals as compared to* Labeo rohita*.

## 1. Introduction

Health and environmental problems arising from heavy metals present in aquatic ecosystem and their bioaccumulation in fishes are very well known [1]. Heavy metals are widely distributed in water and are very essential in trace amount for normal biological and physiological activities of aquatic organisms [2]. Heavy metals have gained much consideration among the nondegradable noxious substance owing to their poor consequences on water inhibiting fauna and flora [3]. Heavy metals are the most toxic substances because of their varied effects. Metals with higher concentration are known to cause harmful effects on the blood and organs of the fish. They form metals compounds when reacting with enzymes, deoxyribonucleic acid, ribonucleic acid, and cellular proteins [4]. Chromium (Cr) in hexavalent form is comparatively active in the surrounding and is extremely toxic which may cause cancer and embryonic defects in aquatic organisms [5]. Cr (V1) compounds are very toxic even at low concentrations but the toxicity depends on pH value of the aquatic body [6]. Yousafzai [7] reported that high concentration of Cr in the gills of* Labeo dyocheilus* and* Wallago attu*. Zinc frequently appears everywhere in the environment. About 70 ppm of Zn is found in different strata of the earth [8]. Zinc also occurs in numerous other forms, including hydrate ions and complex metals [9]. Different anthropogenic activities such as mining, smelting, and use of Zn in dye castings metals, plastic, and alloys can direct its release towards the aquatic ecosystem through various channels and streams [10]. Zinc enters into the fish body through different pathways, including skin or general body surface, gills and alimentary canal, or gut by consumption of contaminated food [11]. The homeostatic level of the body is disturbed when lead is accumulated in the tissue; for example, aminolevulinate (ALA) is one of the intermediate products during heme synthesis. It is condensed by Zn containing enzyme ALA dehydrogenase which is very sensitive to inhibition by lead toxicity [12]. This ALA accumulates in the cell and creates serious problems, that is, neurological disorder, accumulation of lactate in the cell, low pH of the protoplasm, and outflux of calcium from the bone [13]. Essential heavy metals are taken up by fishes and other aquatic organisms from water, food, and sediments [14]. The natural balance of heavy metals concentration in water is highly disturbing with increasing trend of industrial and agricultural activities [15]. The excessive uptake of essential and nonessential heavy metals causes its accumulation in the body organs and tissues [16]. Heavy metals in higher concentration change the physiological and biological activities of the fish [1, 17]. Consumption of such metal contaminated fishes by human can cause serious health issues, Kamaruzzaman et al. [18].

Limnologists are highly concerned about the increasing contaminations of water bodies due to heavy metals which are more dominant in lotic systems towards which industrial wastes are directed. Heavy metals deteriorate the ecological balance of the aquatic environment [1, 19–21]. Because fishes are at the end of aquatic trophic level they have higher tendency to accumulate heavy metals in their body (Jakimiska et al., 2011). In aquatic system they diffuse radially and fish often being on the top of aquatic food chain are more susceptible to the hazardous effects as compared to terrestrial vertebrates [22] (Chezhian et al., 2010) and it is critical to investigate and monitor the bioaccumulation pattern [23]. Heavy metals bioaccumulation in fishes restricts their use as a food due to the threats they pose to health; therefore assessment of fishes of different aquatic habitat for heavy metals accumulation is very much important.

We aimed to investigate heavy metals bioaccumulation in different organs of* Cyprinus carpio* and* Labeo rohita* and to determine which of the species have comparatively higher accumulation of heavy metals from Sardaryab, a tributary of River Kabul.

## 2. Materials and Methods

Fish were collected with the help of cast net from Sardaryab Tapu, River Kabul, one kilometer upstream from Sardaryab Bridge. Morphometric measurements were taken on the spot and then fishes were transported to PCSIR (Pakistan council of scientific and industrial research) laboratory, packed in ice bar in a plastic bag.

In the laboratory fishes were dissected for gills, liver, and muscles with the help of stainless steel knife on a clean glass. The organs were dried in the sun for one day.

The dried organs were weighted and kept in oven at 120°C for one hour and then cooled in a desiccator. One gram (three samples from each organ and each species) from each cooled sample was weighted through electric balance and transferred to beakers of 50 mL capacity thoroughly washed with distilled water. After this 10 mL concentrated HNO_3_ + 20 mL HCl was added to each beaker containing specific organ samples and they were heated gently on a hot plate 200–250°C to digest the samples completely. The hot plate was kept in ventilation hub because of acidic fumes that originate from the samples and was heated until the solution became clear and transparent. The solution was evaporated up to 0.5 mL and until dense white fumes started after the brown fumes. This is an indication that the digestion is completed. The solution was diluted to 10 mL by adding distilled water and rinsing the beaker [24]. The samples were stored in a 15 mL falcon tube for further analysis. The six heavy metals were then determined by using Atomic Absorption Spectrophotometer (Shimadzu AA- 6601). Mean values of the measured concentrations were expressed in *μ*g/g.

## 3. Results

Bioaccumulation of heavy metals Zn, Cr, Cu, Hg, Fe, and Pb in different organs of* Cyprinus carpio* and* Labeo rohita* from Sardaryab River Kabul was investigated in the present study and the results are presented in Table 1. Data was expressed as mean ± standard error of the mean (SEM) with the help of excel software.

In gills of* Cyprinus carpio* the quantity of Zn was 0.074 ± 0.01, Cr was 0.154 ± 0.011, Fe was 0.086 ± 0.008, Cu was 0.024 ± 0.004, and Pb was 0.041 ± 0.019 ug/g. In liver of* Cyprinus carpio* the quantity of Zn was 0.07 ± 0.009, Cr was 0.188 ± 0.007, Fe was 0.08 ± 0.008, Cu was 0.089 ± 0.007, and Pb was 0.142 ± 0.011. In the muscles of the same fish the accumulation of Zn was 0.018 ± 0.004, Cr was 0.024 ± 0.008, Fe was 0.067 ± 0.008, and Cu was 0.016 ± 0.008 *μ*g/g.

Similarly in the gills of* Labeo rohita* the quantity of Zn was 0.058 ± 0.009, Cr was 0.133 ± 0.008, Fe was 0.08 ± 0.01, Cu was 0.018 ± 0.006, and Pb was 0.024 ± 0.014 ug/g. In liver of* Labeo rohita* the quantity of Zn was 0.088 ± 008, Cr was 0.165 ± 0.01, Fe was 0.061 ± 0.011, Cu was 0.071 ± 0.01, and Pb was 0.161 ± 0.011 ug/g, while in the muscles of this fish the storage of Zn was 0.02 ± 0.008, Cr was 0.019 ± 0.006, Fe was 0.05 ± 0.01, and Cu was 0.01 ± 0.004 ug/g.

Overall concentration of the studied heavy metals was higher in* Cyprinus carpio* as compared to* Labeo rohita*. Hg was not detected in neither of the species whereas Pb was not detected in muscles of both species. All the investigated heavy metals were found within the US (RDA) permissible dietary limits. Overall order of heavy metals burden in* Cyprinus carpio* and* Labeo rohita* was found to be as liver > gills > muscles.

## 4. Discussion

Bioaccumulations of heavy metals are used for environmental monitoring largely because aquatic organisms are in direct contact with the contaminated water. Tissue metal concentrations in fish are good indicators of aquatic system exposure to the metal contamination [25, 26]. Heavy metals accumulate in fishes via water, sediments, food such as algae upon which both herbivorous and omnivorous fishes feed [14, 27].

In the present study we found that Cr and Fe were in higher concentrations followed by Zn in different organs of the* Cyprinus carpio*. In addition to common carp higher level of Cr was also detected in* Labeo rohita*. Accumulation pattern of zinc was reported in the same order and was highest in liver of* Labeo rohita* as reported by Palaniappan et al., 2010. All of the heavy metals that we studied here such as Fe, Zn, Cu, Pb, and Cr are also reported by Onwumere and Oladimeji [28] in* Oreochromis niloticus* that were in contact with petroleum refinery effluent. In our findings the accumulation of metals was higher in liver and gills. Livers of both species showed higher concentrations of heavy metals while muscles showed the least amount except iron (Fe) which was highest in gills of* Labeo rohita*. Heavy metals usually mount up in metabolically active tissues [29]. The tissue of liver is extremely active in the storage and uptake of heavy metals and also it is known that metallothionein induction takes place in fish liver [30]. The higher concentration of heavy metals in liver may also be due to the storage and detoxification of heavy metals in liver, coming through food [31, 32]. The accumulation of metals in gills is probably because of direct contact of the metals with the gills during respiration [27]. It has been reported that the accumulation of heavy metals in gills is because of its thinnest epithelium among all the organs of the body through which metals can easily pass [27]. Mastan [21] has also been reported the similar pattern of bioaccumulation in* Labeo rohita*. Experimental studies have shown that muscles of* Labeo rohita* accumulate least metal zinc as compared to other organs [33]. Bioaccumulation of heavy metals like mercury, chromium, and nickel has been documented to be influenced by variation in age, season, and gender and this may correlate with feeding habits in different seasons and areas [34–36]. In a study it has been reported that* Cyprinus carpio* gut content contains 45% detritus [37]; such condition may increase the chances of heavy metals accumulation in the interacting tissues such as stomach and liver. Connection between feeding habits, foraging behavior, and heavy metals concentration is also well established as higher for omnivorous and herbivorous as compared to carnivorous because omnivorous fishes feeding from different food chains are considered to have greater chances of heavy metals bioaccumulation [1, 36, 38]. Thus the omnivore nature of* Cyprinus carpio* makes it more prone to heavy metals bioaccumulation as compared to herbivore* Labeo rohita*. Water having accumulated heavy metals is a source for bioaccumulation in the gills; being the respiratory route they are in direct contact with water [1, 19, 27].

It has been a common trend in most cases that accumulation has been the highest in liver and lowest in muscles. Gills, skin, and alimentary canal are the entry points of heavy metals. Active interaction of the tissue type, that is, gills with contaminated water and liver exposure to contaminated food, is another reason for the concentration of heavy metals in the respective tissues [39]. It has been published that Cr generally does not accumulate in fish and hence low Cr concentrations are reported even from the worldwide industrial areas [40]. In contrast to this study Yousafzai [7] published a report showing that significant Cr was accumulated in the gills of* Labeo dyocheilus* and* Wallago attu*. In addition to it, other studies also reported that higher concentration of Cr is a usual trend of bioaccumulation in different fishes such as* Channa punctatus* [39] and* Wallago attu* [41]. This is probably because the physiology of each fish is susceptible to a specific type of metal.

All the studied heavy metals do not exceed the limits set by US (RDA) (Table 1) on the basis of which it can be said that no immediate risks are there in consumption of the fishes. Long term monitoring may be needed for a true picture in addition to a test for sublethal concentration in particular species.

It is concluded from the current study that* Cyprinus carpio* stored higher concentration of Cr in all three mentioned organs as compared to* Labeo rohita*. Hence* Labeo rohita* is good for consumption as it accumulates least amount of Cr.

## Figures and Tables

**Table 1:** Showing heavy metal concentrations in gills, liver, and muscle of *Cyprinus carpio* and *Labeo rohita*.

Fish species	Tissue	Concentrations (*μ*g/g)					US (RDA) limits
		Cr	Zn	Fe	Cu	Pb	Cr 11–45 *μ*g
*Cyprinus carpio*	Gills	0.154 ± 0.011	0.074 ± 0.01	0.086 ± 0.008	0.024 ± 0.004	0.041 ± 0.019	Zn 2600 *μ*g
	Liver	0.188 ± 0.007	0.07 ± 0.009	0.08 ± 0.008	0.089 ± 0.007	0.142 ± 0.011	Fe
	Muscles	0.024 ± 0.008	0.018 ± 0.004	0.067 ± 0.008	0.016 ± 0.008	0	Cu 2000–3000 *μ*g

*Labeo rohita*	Gills	0.133 ± 0.008	0.058 ± 0.009	0.08 ± 0.01	0.018 ± 0.006	0.024 ± 0.014	Pb 300 *μ*g
	Liver	0.165 ± 0.01	0.088 ± 0.008	0.061 ± 0.011	0.071 ± 0.01	0.161 ± 0.011	
	Muscles	0.019 ± 0.006	0.02 ± 0.008	0.05 ± 0.01	0.01 ± 0.004	0	
